# Reconsidering the policy options to combat obesity

**DOI:** 10.1186/1753-6561-6-S3-O5

**Published:** 2012-06-01

**Authors:** Christine Ferguson

**Affiliations:** 1 School of Public Health and Health Services, Department of Health Policy, George Washington University, 2021 K St, NW Suite 800, Washington, DC 20006, USA

## 

As the prevalence of obesity steadily grows, so too does our understanding of the threats that excess weight poses to both our individual health and our nation as a whole. With this information, there is a need now more than ever to reconsider the options we have to mitigate the health risks and costs of this growing epidemic. This includes, among other conversations, examining how employers can play a role in encouraging healthier habits through workforce-based initiatives and a reconsideration of the risk-benefit paradigm that informs the decisions of payers, regulators, and providers on this crucial issue.

